# Efficacy and safety of *Ginkgo biloba* for patients with early diabetic nephropathy: A protocol for systematic review and meta-analysis

**DOI:** 10.1097/MD.0000000000021959

**Published:** 2020-08-28

**Authors:** Hongyun Wang, Meilin Yuan, Xinrong Zou

**Affiliations:** a Clinical College of Traditional Chinese Medicine, Hubei University of Chinese Medicine; b Department of Nephrology, Hubei Provincial Hospital of Traditional Chinese Medicine; c Hubei Province Academy of Traditional Chinese Medicine, Wuhan, Hubei Province, China.

**Keywords:** diabetic nephropathy, *Ginkgo biloba*, meta-analysis, protocol, systematic review

## Abstract

**Background:**

Diabetic nephropathy (DN) is not only an important microvascular complication of diabetes but also the main cause of end-stage renal disease. *Ginkgo biloba* has a variety of biological activities and has been widely used in China to treat kidney diseases such as DN. This article aimed to evaluate the efficacy and safety of *G biloba* in patients affected with DN in the early stage.

**Methods:**

This protocol follows the preferred reporting items for systematic review and meta-analysis protocols and the recommendations of the Cochrane Collaboration Handbook. Seven electronic databases will be searched from inception to July 31, 2020. Two investigators will independently identify relevant randomized controlled trials, fetch data, and assess the risk of bias with tools provided by Cochrane. A comprehensive meta-analysis will be conducted with the Cochrane Collaboration software (Review Manager 5.3) for eligible and appropriate studies. Further, the evidence will be assessed with the Grading of Recommendations Assessment, Development, and Evaluation approach.

**Results:**

The results will be published in academic peer-reviewed journals, and the evidence gathered by this project will be dedicated to assessing the efficacy and safety of *G biloba* for DN patients in the early stage.

**Conclusion:**

This systematic review and meta-analysis will synthesize the available evidence to demonstrate the efficacy of *G biloba* in delaying the progression of patients with early DN.

**Trial registration number:**

PROSPERO CRD42020166805.

## 1 Introduction

Diabetic Nephropathy (DN), the most common microvascular complication of diabetes, is characterized by persistent microalbuminuria, renal tubular, and interstitial fibrosis, which is also the most common etiology of end-stage renal disease.^[1,2]^ The incidence of DN ranges from 25% to 40% in type 1 diabetic patients to 5% to 40% in type 2 diabetic patients.^[3]^ DN is a progressive process. Its early clinical manifestations are glomerular hyperfiltration and increased urinary albumin excretion rate. Its pathological features are glomerular basement membrane thickening, mesangial dilatation, and tuberous sclerosis.^[4,5]^ With the development of DN, the number of damaged glomeruli increases and the glomerular filtration rate decreases significantly. The clinical manifestations of this stage are massive proteinuria and glomerular and tubulointerstitial fibrosis.^[6,7]^ According to Mogensen stage,^[8,9]^ DN can be divided into 5 stages. Due to the imperceptible symptoms of stage I and II, most patients of DN were not diagnosed until stage III or after stage III.^[10]^ Once DN enters its end stage (stage V), treatment will be more difficult than other kidney diseases.^[11]^ Thus, it becomes particularly important to find interventions that can delay the progression of DN in the early stage. At present, the key to the treatment of DN is to strengthen blood glucose control, so new anti-diabetic drugs with specific renal protective effects are widely used in clinical practice.^[12]^ In addition, previous studies have identified 2 major kinds of antihypertensive drugs, that is, angiotensin-converting enzyme inhibitors and angiotensin receptor blockers, which can slow down the progression of kidney disease by controlling albuminuria.^[13]^ Unfortunately, despite tremendous advances in the pharmaceutical industry, no new therapies that specifically improve the progression of DN have been successfully applied in the clinic.^[14]^ For the above reasons, there is an urgent need to find a novel and effective therapy to intervene in controlling the progress of DN, especially in the early stage.

*Ginkgo biloba*, a traditional Chinese herbal medicine, is one of the most widely used phytotherapeutic products in the world, with thousands of years of clinical application history.^[15]^ There is evidence that *G biloba* may have potential therapeutic effects on a variety of diseases, such as diabetic cardiomyopathy, neurodegenerative diseases, neurodegenerative retinal diseases, myocardial lesion, hippocampus neuronal lesions, cancer, obesity, and liver damage.^[16–20]^ Modern pharmacological studies have revealed that it has antioxidant, anti-inflammatory,^[21]^ neuroprotective,^[22]^ and anti-platelet aggregation effects.^[23]^ Flavonol glycosides and terpene trilactones are the main components to exert biological activities.^[24]^ The preventive and therapeutic effects of *G biloba* on DN have been confirmed by several animal experiments.^[25–27]^*G biloba* can reverse the increase of fasting blood glucose, 24 hours urinary protein, blood urea nitrogen, and creatinine and improve the change of renal ultrastructure in DN rats.^[28]^ According to the results of a metabolomic study, oleic acid and glutamate may be the potential biomarkers for *G biloba* against kidney injury.^[28]^ Besides, *G biloba* can also reduce the expression of E-cadherin, alpha-smooth muscle actin, snail, and the phosphorylation levels of AKT, mTOR, and p70S6K in the diabetic renal cortex suggesting that *G biloba* may prevent renal fibrosis in DN rats by inhibiting the Akt/mTOR signaling pathway.^[29]^ In a randomized, double-blind, multi-center, controlled trial, researchers found that *G biloba* could attenuate deterioration of albuminuria in type 2 diabetes patients, which indicating that *G biloba* could be a promising option of renoprotective agents for the early stage of DN.^[30]^

This paper aims to update and summarize the efficacy and safety of *G biloba* in the treatment of early DN, and to provide reliable evidence for clinical practice and further study of early DN.

## 2 Methods

### 2.1 Protocol register

This study protocol was drafted under the guidance of the preferred reporting items for systematic reviews and meta-analyses protocols statement.^[31]^ The study was previously registered on the PROSPERO (https://www.crd.york.ac.uk/PROSPERO/), and a registration number has been assigned: CRD 42020166805.

### 2.2 Ethics

Ethical approval will not be required because data included in this meta-analysis will be extracted from published trials that meet accepted ethical standards.

### 2.3 Database search strategy

We will search the following databases from their inception to July 31, 2020: China National Knowledge Infrastructure, Chinese Scientific Journals Database, Wanfang Database, China Biological Medicine Database, PubMed, EMBASE Database and Cochrane Central Register of Controlled Trials. Languages will be limited to Chinese or English. We will search the reference lists of the identified trials to identify further relevant trials. We will also search online trial registries such as ClinicalTrials.gov (Clinical Trials.gov/), European Medicines Agency (www.ema.europa.eu/ema/), World Health Organization International Clinical Trials Registry Platform (www.who.int/ictrp), the Food and Drug Administration (www.fda.gov), as well as pharmaceutical company sources for ongoing or unpublished trials. The specific search strategy will be formulated with the specific database. Among them, the author lists the retrieval strategies of the PubMed database (see Table 1). It will also be supplemented by a manual search of relevant literature. Computer retrieval and manual retrieval will be utilized to retrieve all the published literature by 2 authors (Hongyun Wang and Meilin Yuan), respectively.

**Table 1:**
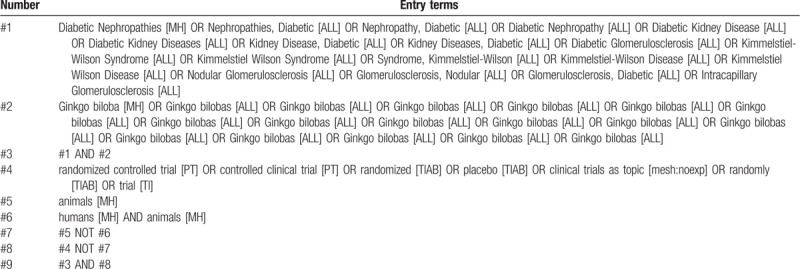
Search strategy for PubMed.

### 2.4 Eligibility criteria and elimination criteria

#### 2.4.1 Types of participants

Studies recruiting patients with early DN, as diagnosed with any recognized diagnostic criteria, will be included in our study, regardless of gender, ethnic background or nationality. However, subjects with infection, fever, cancer, kidney transplantation, liver disease, and severe cardiopulmonary disease will be excluded.

#### 2.4.2 Types of interventions

Interventions assigned to the experimental groups must be *G biloba*. The intervention in the control group could be routine treatment refers to diet, blood sugar, blood pressure, anticoagulation, and other positive intervention drugs. Positive drugs, such as calcium ion antagonists, can be used in both groups but should be consistent between the 2 groups. The dose, dosage form, and treatment duration of the 2 groups will not be taken into considerations in this study.

#### 2.4.3 Types of outcome measures

The primary outcome measures set in our study included urinary albumin excretion rates, 24 hours urinary protein quantification, and renal function (blood urea nitrogen, serum creatinine concentration). Correspondingly, blood pressure, blood lipids, blood glucose, hemorheology, and adverse events were set as secondary outcome measures. All outcomes were collected from the beginning of the experiment to the last available follow-up.

#### 2.4.4 Type of study

Randomized controlled trials using *G biloba* as an intervention for patients with early DN will be included, regardless of whether the trials adopted the blind method or not. It should be noted that all quasi-randomized controlled trials recruiting volunteers according to medical record number or birthday will not be considered for further screening.

### 2.5 Study selection and data collection

**Figure 1:**
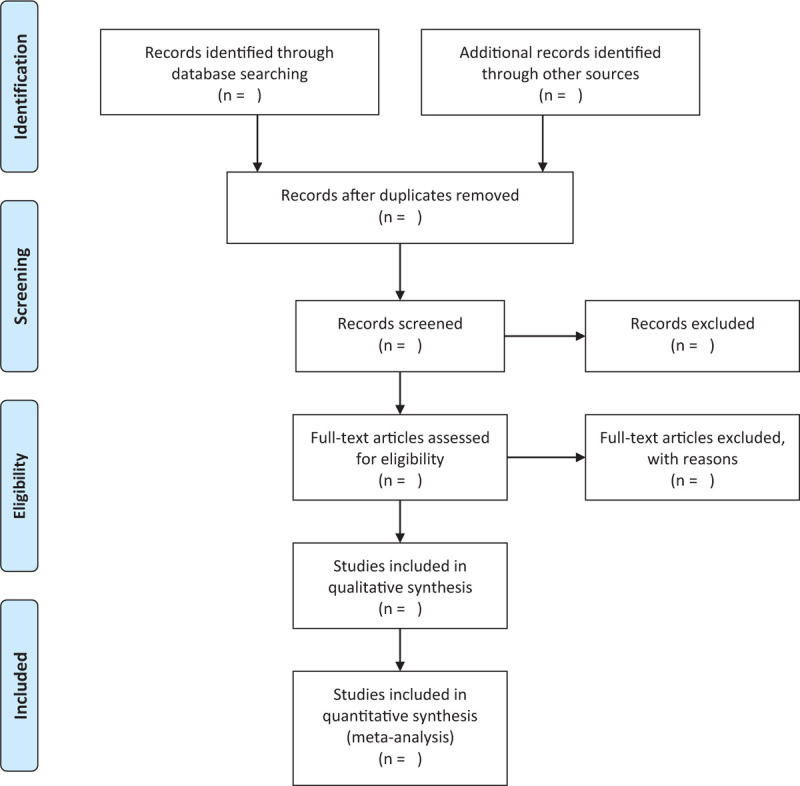
The flow chart of study selection process.

The above collected literature will be managed by a reference manage software. Two independent reviewers (Wang Hongyun and Yuan Meilin) will review and select studies according to the above qualification criteria, and then cross check the screening results. Any discrepancies will be discussed with the third reviewer (Zou Xinrong) for resolution. The flow chart of screening is shown in Figure 1. A pre-designed data extraction table will be utilized to extract the data, as follows:

(1) basic characteristics of the literature, including the title, year, source, author and contact information, and so on;
(2) research methods: including research design, random units, random methods, covert grouping, blinding, duration, and so on;
(3) relevant characteristics of the study subjects, including number, age, gender, and so on;
(4) intervention measures and control measures, including specific methods, number of people, route of administration, dosage, time, course of treatment, follow-up, and so on;
(5) outcome indicators and measurement methods;
(6) results.

If the information in the text is incomplete, the original study author will be contacted to obtain the necessary data.

### 2.6 Literature quality assessment

Two investigators (Hongyun Wang and Meilin Yuan) will independently assess the risk of bias in included studies using the Cochrane risk of bias tool,^[32]^ which considers the following 7 aspects: random sequence generation, allocation concealment, blinding of participants and personnel, blinding of outcome assessment, incomplete outcome data, selective reporting, and other bias. The score of each article is distributed from 0 to 7. Disagreements between the review authors over the risk of bias in particular studies will be resolved by discussion, with the involvement of a third review author (Xinrong Zou) where necessary.

### 2.7 Statistical analysis

The statistical analysis will be performed by implementing the Cochrane Collaboration Review Manager software (RevMan5.3). Dichotomous data will be analyzed by using the risk ratio with 95% confidence interval, and continuous variables will be analyzed by using the mean difference with 95% confidence interval. The *I*^2^ statistic will be calculated to value the heterogeneity among studies. The heterogeneity can be expected as statistically significant if the *I*^2^ statistic are more than 50%. The presence or absence of significant heterogeneity decided the option of a random-effects model or fixed-effects model. If necessary, subgroup analyses may be done according to the duration of treatment, or the dosage, or the intervention. Sensitivity analysis will be carried out by omitting 1 study at a time. Only when a subgroup includes 10 or more studies will publication bias be measured.

### 2.8 Grading of recommendations assessment, development, and evaluation quality assessment

This assessment will be conducted with the Guideline Development Tool (https://gradepro.org/). On account of the grading of recommendations assessment, development, and evaluation handbook,^[33]^ 2 investigators will respectively classify the quality of evidence into the following 4 levels: high quality, moderate quality, low quality, and very low quality.

## 3 Discussion

A previous article has systematically reviewed and confirmed the efficacy and safety of *G biloba* for early DN.^[34]^ However, due to the deficiency of the methodological quality of the included studies, there is still a lack of strong clinical evidence. In addition, a number of new clinical trials have been carried out in recent years, especially in China. Thus, it is necessary to conduct a new systematic evaluation again to provide convincing evidence on the effectiveness and safety of *G biloba* for DN in the early stage.

It must be recognized that certain restrictions may affect conclusions drawn under this protocol. The retrieved studies only included those published in Chinese or English. If some differences in diagnostic criteria, intervention measures, dosage, and duration of medication and so forth cannot be properly resolved, it may lead to excessive heterogeneity, which will affect the reliability of the conclusion.

## Author contributions

**Conceptualization:** Xinrong Zou and Hongyun Wang.

**Data curation:** Hongyun Wang and Meilin Yuan.

**Formal analysis:** Hongyun Wang and Meilin Yuan.

**Methodology:** Hongyun Wang and Meilin Yuan.

**Project administration:** Xinrong Zou.

**Supervision:** Xinrong Zou.

**Validation:** Xinrong Zou.

**Writing – original draft:** Hongyun Wang, Meilin Yuan, and Xinrong Zou.
